# Redefining Success in Oral and Maxillofacial Surgery: Bridging the Gap Between Technical Outcomes and Patient Quality of Life

**DOI:** 10.1002/snz2.70072

**Published:** 2026-08-05

**Authors:** Betul Gedik, Mehmet Ali Erdem, Abdulkadir Burak Cankaya, Zohaib Khurshid

**Affiliations:** ^1^ Faculty of Dentistry Department of Oral and Maxillofacial Surgery Istanbul University Istanbul Turkey; ^2^ College of Dentistry Department of Prosthodontics and Dental Implantology King Faisal University Al‐Ahsa Saudi Arabia; ^3^ Faculty of Dentistry Department of Anatomy Center of Artificial Intelligence and Innovation (CAII) Center of Excellence for Dental Stem Cell Biology Chulalongkorn University Bangkok Thailand; ^4^ Department of Oral and Maxillofacial Surgery Biruni University Istanbul Turkey

**Keywords:** dental implantology, oral and maxillofacial surgery, orthognathic surgery, quality of life, temporomandibular disorders

## Abstract

Traditional success metrics in oral and maxillofacial surgery (OMFS) primarily emphasize clinician‐reported outcomes such as anatomical correction, osseointegration, skeletal stability, and disease control. However, growing evidence indicates a frequent mismatch between these objective measures and patient‐reported outcome measures (PROMs) and quality of life (QoL), particularly in interventions affecting facial appearance and socially relevant functions. This narrative review examines the alignment and divergence between clinical outcomes and PROMs across major OMFS subspecialties, including dental implantology, orthognathic surgery, temporomandibular joint surgery, and oral cancer reconstruction. Across these domains, technical success often fails to translate into perceived patient benefit, with persistent functional, esthetic, or psychosocial concerns despite clinical stability. These findings underscore the need to redefine success in OMFS beyond structural restoration. Incorporating standardized PROMs as coprimary outcomes and adopting patient‐centered, multidisciplinary care models are essential for achieving outcomes that reflect both clinical efficacy and meaningful improvements in patients’ lived experiences.

## 1 Introduction

Oral and maxillofacial surgery (OMFS) encompasses procedures that directly influence core human functions including chewing, swallowing, speaking, and facial esthetics. Historically, the success of these procedures has been primarily judged by clinician‐reported outcomes, rooted in anatomical restoration, functional recovery assessed through clinical examination, radiographic measurements, absence of complications, and prosthesis survival statistics (Rutkowski et al. 2022). These metrics have guided research, funding priorities, and training over decades, and they remain indispensable for establishing procedural feasibility and safety.

However, there is growing recognition that patient experiences and perceived benefits do not always align with clinically measured “success.” The shift toward value‐based healthcare places greater emphasis not only on extending life or restoring anatomy but also on improving how life is lived after treatment (Gangannagaripalli et al. 2022). This shift is particularly relevant in OMFS, where many procedures, including orthognathic surgery and head and neck cancer (HNC) reconstruction, profoundly influence psychosocial identity, interpersonal interaction, and overall well‐being. As a result, patient‐reported outcome measures (PROMs) such as the Oral Health Impact Profile (OHIP‐14), Orthognathic Quality of Life Questionnaire (OQLQ), FACE‐Q, University of Washington Quality of Life (UW‐QoL), and EORTC QLQ‐H&N35 have become essential tools for capturing the patient's lived experience (Tachiki et al. 2018).

Despite increasing implementation, substantive gaps and mismatches persist between clinician‐based success definitions and PROM outcomes across various OMFS subspecialties. For example, in implantology, radiographically stable implants may coexist with ongoing speech or esthetic dissatisfaction. After orthognathic surgery, patients may achieve cephalometric perfection yet remain unhappy with self‐image. HNC survivors frequently struggle with communication, nutrition, and social withdrawal long after oncologic cure has been achieved.

Therefore, understanding the convergence and divergence of clinical outcomes and PROMs—and identifying the reasons behind these discrepancies—is critical for redefining success in OMFS. This review critically evaluates outcome interpretation in the major OMFS domains, identifies where outcome definitions fail to align with patient expectations, and proposes a holistic, patient‐centered framework that better represents the actual therapeutic benefit experienced by patients. It was based on a literature search of PubMed, Scopus, and Web of Science databases through December 2025. Search terms included combinations of “oral and maxillofacial surgery,” “patient‐reported outcomes,” “quality of life,” “dental implants,” “orthognathic surgery,” “TMJ surgery,” and “head and neck cancer.” English‐language studies relevant to PROMs and quality‐of‐life outcomes in OMFS were considered, and additional articles were identified through manual screening of reference lists. As a narrative review, no formal risk‐of‐bias assessment was performed.

## 2 Dental Implantology and Prosthetic Rehabilitation

Dental implants are widely regarded as one of the most successful rehabilitative interventions in dentistry, achieving survival rates exceeding 90%–98% in long‐term follow‐up studies (French et al. 2021). Clinicians traditionally define implant success based on objective criteria such as implant stability, marginal bone maintenance, absence of biological/prosthetic complications, and strong osseointegration (Gadzo et al. 2023). These metrics, while crucial, are limited to biomechanical behavior and structural integrity. They cannot reflect whether the implant truly restores the functional efficiency, esthetics, and psychological comfort a patient expects when choosing implant treatment.

From a patient perspective, the implant experience is inherently multidimensional. A patient receiving anterior maxillary implants may primarily value esthetic appearance and a confident smile, whereas an edentulous mandibular overdenture patient prioritizes improved chewing ability and speech clarity. Studies repeatedly show that implants do not automatically guarantee high QoL, even when clinically successful (Rella et al. 2023). Dissatisfaction may arise from soft‐tissue disharmony, unnatural smile contours, phonetic difficulties, food entrapment, or fear of implant failure—domains rarely captured in clinical studies (Wang et al. 2021).

Psychosocial determinants add further complexity. Patients with high preoperative expectations may perceive moderate esthetic improvements as failures despite excellent technical outcomes. Conversely, lower‐expectation patients may report remarkable improvements even when minor clinical imperfections remain. The strong influence of expectation alignment underscores the value of shared decision‐making and digital visualization tools in implant planning.

A consistent theme in the literature is the time‐dependent nature of PROM improvement. In the first 3 months postloading, adaptation challenges may cause transient declines in QoL scores. Over time, typically 6–12 months, functional gains become more noticeable, and response‐shift phenomena may recalibrate patient expectations, improving subjective satisfaction regardless of clinical change (Machuca et al. 2020). This confirms that single‐time‐point PROM measurements are inadequate.

These insights reveal that implant survival ≠ treatment success. True success must incorporate the actual benefit patients feel in their everyday lives. PROMs should therefore shift from being secondary outcomes to coprimary endpoints in implant studies. Table 1 summarizes the differences between clinical and PROM priorities in implant treatment.

**TABLE 1:** Comparison of clinical and PROM priorities in implantology.

Dimension	Clinician priority	Patient priority
Biological outcomes	Bone level stability	Absence of pain and infection
Functional outcomes	Proper occlusion	Improved chewing and speaking
Esthetics	Tissue integration	Natural smile, self‐confidence
Psychosocial	Rarely considered	Social acceptance and quality of life

## 3 Orthognathic Surgery and Dentofacial Deformities

Orthognathic surgery is a complex intervention aimed at restoring both dental occlusion and facial harmony in patients with dentofacial deformities. In clinical practice, surgeons mainly assess outcomes through cephalometric changes, dental arch coordination, relapse rates, neurosensory recovery, and complication profiles (Benington et al. 2024). These measures are critical for verifying surgical precision and skeletal stability. For instance, the normalization of ANB angles to reflect skeletal Class I occlusion is a typical clinical measure of success. Persistent lower lip or chin paresthesia may also negatively affect patient satisfaction despite otherwise successful surgical outcomes, highlighting the importance of neurosensory recovery as a patient‐centered outcome measure. However, these objective endpoints do not fully capture whether patients experience the improvements in self‐confidence, social comfort, and functional convenience that often motivate surgery in the first place.

A principal challenge in orthognathic outcome evaluation lies in its highly psychosocial purpose. Unlike orthopedics or other reconstructive fields where functional recovery dominates, a significant proportion of patients pursue orthognathic surgery primarily for esthetic and psychological enhancements, including reduction in social anxiety, improvement in interpersonal interactions, and enhancement of perceived attractiveness (Liddle et al. 2018). As such, dissatisfaction may persist even when occlusion is technically ideal, especially if subtle asymmetries remain or if the final facial appearance does not align with preoperative expectations. This underscores the necessity of evaluating subjective self‐image and patient satisfaction as primary success markers.

The trajectory of postoperative QoL is also unique. The early healing phase (first 6–8 weeks) may result in a decline in QoL related to chewing limitations, postoperative pain, and restricted social activity. During this time, PROM scores such as OQLQ are expected to show temporary deterioration. Over the subsequent 6–12 months, patients typically report substantial improvements in esthetic satisfaction, facial function, and social participation, indicating a longitudinal recovery pattern that differs significantly from the immediate clinical indicators. Several studies demonstrate that the psychosocial benefits of orthognathic surgery continue to increase even after technical stability plateaus, suggesting that long‐term PROM assessments are essential to accurately capture success (Alanko et al. 2017). This continued improvement may reflect gradual social reintegration, increased self‐confidence, adaptation to facial appearance changes, and growing satisfaction as patients become accustomed to their postoperative identity.

Expectation misalignment is one of the strongest predictors of discordance between clinician‐ and patient‐perceived outcomes. Patients presenting with severe social anxiety or unrealistic expectations—often fueled by social media esthetic ideals—require careful psychological preparation prior to surgery (Taloumtzi et al. 2021). Digital simulation and virtual planning tools can significantly enhance expectation alignment, fostering shared decision‐making and improved satisfaction. Furthermore, sex differences exist: Female patients typically prioritize facial esthetics more strongly, whereas male patients report more value from functional gains like chewing efficiency (Stagles et al. 2016). The comparison of clinical and patient‐centered priorities in orthognathic surgery is shown in Table 2.

**TABLE 2:** Clinical vs. patient‐centered priorities in orthognathic surgery.

Outcome domain	Clinician‐relevant goals	Patient‐relevant goals
Skeletal stability	Cephalometric normalization, relapse prevention	Harmonized facial profile and symmetry
Dental function	Achieved Class I occlusion, mastication mechanics	Chewing comfort, speech improvement
Esthetics	Improved soft‐tissue contour	Attractive smile, natural appearance, social acceptance
Psychosocial outcomes	Rarely measured	Increased confidence, reduced social anxiety
Sensory outcomes	Nerve recovery	Fear of permanent numbness, sensory comfort

Overall, orthognathic surgery represents the most prominent example in OMFS where technical perfection does not guarantee patient satisfaction. Surgical success in this field must therefore be defined not only through skeletal and occlusal outcomes but also through the holistic restoration of self‐image, social ease, and daily functional well‐being. PROMs should not be supplementary metrics but rather central indicators guiding treatment planning, perioperative counseling, and long‐term outcome studies.

## 4 Temporomandibular Joint (TMJ) Surgery and Reconstruction

Temporomandibular joint disorders (TMDs) significantly affect daily functioning by limiting mandibular mobility, causing persistent pain, and restricting nutrition and social behavior. When conservative treatment fails, TMJ surgery—including arthrocentesis, arthroscopy, disc repositioning procedures, and total joint replacement (TMJR)—may be indicated (Murphy et al. 2013). Traditionally, surgical success has been evaluated through maximum mouth opening measurements, radiographic stability, and reduction in clicking or locking events. Although such metrics reflect biomechanical improvements, they do not consistently align with patient‐reported relief, especially when chronic pain and psychosocial distress predominate (Bäckström et al. 2024).

One of the most challenging aspects of TMJ surgery is that pain is often the primary complaint, yet pain is inherently subjective and influenced by psychological, hormonal, and behavioral variables. Even with clear gains in mandibular mobility—for instance, increasing mouth opening beyond 35–40 mm—patients frequently continue to report functional discomfort or difficulty with chewing hard foods (Hoa et al. 2025). These residual symptoms can lead to disappointment when expectations were centered on complete pain elimination rather than functional improvement. Thus, clinicians must recognize that pain improvement and functional improvement are not interchangeable outcomes.

Another crucial determinant of discordance is that TMDs often exist within a biopsychosocial framework. Patients with high levels of anxiety, depression, catastrophizing tendencies, or sleep disturbances tend to report poorer postoperative outcomes, regardless of objective findings. TMJDs are also more prevalent in women, and hormonal fluctuations may contribute to persistent symptoms after surgery (Vinayak et al. 2024). Therefore, evaluation of surgical success should incorporate psychological well‐being and coping strategies rather than assuming that anatomic correction alone yields full QoL recovery.

Among surgical treatments, TMJ total joint replacement (TMJR) demonstrates some of the highest postoperative improvements in function, pain reduction, and mastication (Yoda et al. 2020). However, long‐term studies reveal that a subset of patients maintain low emotional well‐being, fear of implant failure, or body image concerns related to facial asymmetry or scarring. In these cases, patients qualitatively report “improved chewing but unchanged life,” which illustrates how QoL is influenced by a broader sense of self‐efficacy and social identity beyond raw functional gains (Mamidi et al. 2019).

Overall, TMJ surgery teaches one of the most important lessons of patient‐centered care: Technical success does not guarantee meaningful recovery (Table 3). Addressing chronic pain mechanisms, expectation management, and psychosocial contributors must be considered core components of therapy. PROMs specific to TMJ function and quality of life should therefore be routinely included in both clinical practice and research protocols—not as secondary endpoints but as primary indicators of true therapeutic benefit.

**TABLE 3:** Clinical vs. patient‐reported outcomes in TMJ surgery.

Outcome dimension	What the surgeon measures	What the patient expects/feels
Functional mobility	Mouth opening ≥35–40 mm	Comfortable eating, speaking without fatigue
Pain	Reduced VAS score	Pain‐free lifestyle, sleep quality restoration
Joint integrity	Stable prosthesis, imaging improvements	Trust in jaw movement, decreased fear of locking
Esthetics	Minimal external deformity	Confidence in facial appearance, reduced self‐consciousness
Psychosocial well‐being	Seldom formally evaluated	Ability to socialize, emotional relief, reduced anxiety

## 5 Oral Cancer Surgery and Reconstruction

HNC, particularly oral cavity and oropharyngeal malignancies, profoundly affects essential functions such as speech, swallowing, mastication, salivation, and facial expression. Surgical treatment—often combined with microvascular reconstruction, radiotherapy, and chemotherapy—is typically successful in achieving oncologic control (Bulsara et al. 2018). Clinician‐defined success focuses on clear surgical margins, absence of local recurrence, stable reconstructive flaps, and acceptable airway protection (Mesia et al. 2021). These metrics are critical from a survival standpoint; however, they evaluate only the biological eradication of disease, not the patient's post‐treatment ability to reintegrate into daily life or maintain personal dignity and autonomy.

Despite optimal oncologic and reconstructive outcomes, HNC survivors frequently continue to experience long‐term functional limitations. Speech intelligibility may remain compromised due to altered tongue biomechanics, velopharyngeal dysfunction, or changes in oral cavity resonance. Dysphagia—ranging from dietary restrictions to gastrostomy dependence—is one of the most debilitating functional sequelae. Additionally, xerostomia and chronic mucosal discomfort caused by radiotherapy can lead to nutritional decline and significantly lower oral health‐related QoL (Kelly and Husain 2016). These limitations often result in a profound impact on social participation, including avoidance of dining in public and isolation from previously enjoyed activities.

Beyond functional impairment, psychosocial well‐being and self‐image frequently deteriorate after HNC treatment. Tumors of the oral cavity alter visible structures associated with identity and expression. Even with meticulous reconstructive effort, scarring, asymmetry, or defects in soft‐tissue contour can negatively affect self‐esteem and social confidence. Patients may report persistent fear of recurrence, stigmatization, or difficulties resuming professional roles (Zebolsky et al. 2021). The more visible the facial disfigurement, the greater the emotional burden, highlighting the importance of esthetic and psychosocial rehabilitation alongside medical management.

PROMs such as the UW‐QoL and EORTC QLQ‐C30/H&N35 have demonstrated that global QoL in HNC survivors remains significantly below population norms even years after treatment (Taylor et al. 2023). Improvements in survival statistics alone therefore do not reflect the lived outcomes of this growing patient population. The literature consistently emphasizes that multidisciplinary rehabilitation—including speech–language therapy, psychological support, nutritional guidance, prosthetic reconstruction, and social reintegration programs—is crucial to restoring meaningful quality of life following cancer surgery (Barrera‐Franco et al. 2017) (Table 4).

**TABLE 4:** Sources of clinician‐ vs. patient‐perceived discordance after oral cancer surgery.

Domain	Clinician perspective	Patient perspective
Oncologic status	“Cancer‐free” = success	Fear of recurrence persists despite remission
Reconstruction	Flap survival = positive outcome	Persistent functional or esthetic deficits
Swallowing	Oral intake achieved	Food avoidance, choking anxiety, diet limitation
Speech	Intelligible speech regained	Embarrassment from altered voice/speech patterns
Esthetics	Acceptable contour/scarring	Loss of identity, negative self‐image
Social life	Clinically stable → discharge	Social withdrawal due to stigma and isolation

Overall, oral cancer surgery reinforces a core message of modern OMFS: Survival alone cannot define success. Once the threat of mortality subsides, patients shift focus to the challenges of living with the consequences of treatment. QoL‐driven care pathways must therefore be integrated from the earliest preoperative phase through survivorship. Achieving true success requires not only removing cancer but also helping individuals reclaim their humanity, confidence, and social belonging.

## 6 Discussion

The findings of this review indicate that OMFS is at a critical turning point in how success should be conceptualized and measured. Historically, the field has relied on clinical indicators—osseointegration, occlusal correction, free‐flap viability, or tumor‐free status—as endpoints believed to reflect optimal outcomes. However, these measures originate from a biomedical paradigm that assumes restoration of anatomy and primary function automatically leads to restored well‐being. The accumulating evidence across implantology, orthognathic surgery, TMJ surgery, and oncologic reconstruction consistently reveals that this assumption is incomplete. Patients may physically survive and function yet fail to recover the essence of life quality, including confidence, autonomy, social belonging, and emotional stability (Zhang et al. 2024). Thus, the discipline is compelled to question not only how well we can operate but what our surgeries truly give back to patients.

Although quantitative estimates vary considerably across studies and indications, the literature consistently demonstrates that clinically successful outcomes do not invariably translate into optimal patient‐reported outcomes. For example, high implant survival rates frequently coexist with persistent esthetic, functional, or psychosocial concerns in a subset of patients. Similarly, substantial improvements in PROM scores following orthognathic surgery, TMJ surgery, and oncologic reconstruction do not necessarily eliminate residual dissatisfaction or unmet expectations. These findings highlight the importance of considering both statistical and clinically meaningful changes, including minimal clinically important differences (MCIDs), when interpreting treatment outcomes.

A major driver of the clinician–patient outcome gap lies in the symbolic and social meaning of the orofacial complex. The face is a bearer of identity, emotion, beauty standards, and social communication (Hernández‐Alfaro and Valls‐Ontañón 2023). Any intervention involving the face therefore produces effects extending far beyond clinical healing trajectories. This becomes particularly visible in orthognathic patients who seek identity transformation in parallel with functional correction. Their judgment of success is influenced by changes in attractiveness, facial harmony, interpersonal treatment, and even employment or relationship opportunities (Honma et al. 2024). The fact that cephalometric perfection may still coexist with self‐consciousness demonstrates that technical excellence does not guarantee social functionality, a domain vastly underestimated in previous generations of OMFS research.

Furthermore, the neurobiological complexity of conditions such as TMD highlights that subjective symptom trajectories do not necessarily mirror objective mechanical improvements. Pain processing involves central sensitization, catastrophization behavior, and emotional modulation (Minye 2020). When surgery does not address these pathways, patients may continue to perceive disability, despite radiological and functional normalization. Hence, psychological screening and cognitive‐behavioral pain management should not be considered adjunctive, but rather fundamental components of TMJ surgical care.

In oncologic surgery, the disconnect is most profound. Here, survival marks only the beginning of a long struggle to reestablish intelligible speech, adequate nutrition, and socially acceptable appearance (Kolokythas 2010). Achieving tumor‐free margins confers clinical satisfaction, but patients may describe themselves as “cancer survivors yet socially invisible.” Persistent fear of recurrence, economic burden, and loss of professional and family roles further deteriorate psychosocial well‐being (Douglas et al. 2023). Importantly, the factors driving postoperative quality of life extend into survivorship care, far beyond the narrow perioperative window traditionally evaluated in OMFS outcome studies.

A key limitation of current outcome science is temporal oversimplification. Many clinical studies report outcomes at 3 or 6 months—points in time where pain and edema may be resolving, but psychological reconstruction has barely begun (Chen et al. 2022). Adaptation to a new appearance, altered speech, and changed dietary routines can take years, and late effects of radiation may only emerge after 24 months. Thus, the absence of long‐term PROM assessments masks the true arc of recovery and leads to premature declarations of “successful outcomes.” Future research must adopt ≥2–5‐year longitudinal designs to fully capture the recovery trajectory and uncover late‐phase declines that early assessments miss.

Another limitation is the heterogeneity in the PROM selection. Tools like OHIP‐14, OQLQ, UW‐QoL, and FACE‐Q assess different constructs; using them interchangeably diminishes cross‐study comparability and weakens meta‐analytic power (Terheyden et al. 2024). Progress requires standardized, indication‐specific PROM frameworks mapped to consistent domains of function, esthetics, emotion, and social participation (Mesia et al. 2021).

An additional challenge in PROM implementation is the selection of appropriate instruments for specific OMFS indications. Commonly used tools differ in their scope, psychometric characteristics, and clinical applicability (Table 5). Generic oral health measures such as OHIP‐14 are widely validated and practical for routine use, whereas condition‐specific instruments including OQLQ, FACE‐Q, UW‐QoL, and EORTC QLQ‐H&N35 provide greater sensitivity to treatment‐related changes within particular patient populations. Therefore, PROM selection should be guided by the clinical context, outcome domains of interest, and intended use in research or clinical practice. Additionally, current PROMs are static measures and do not reflect response‐shift phenomena, where changes in internal expectations alter how patients perceive disability over time (Kelly and Husain 2016). Response shift refers to changes in an individual's internal standards, values, or conceptualization of quality of life following a significant health intervention or life event. According to the theoretical framework proposed by Sprangers and Schwartz, patients may adapt to functional limitations, altered appearance, or chronic symptoms over time, resulting in stable or improved QoL scores despite persistent objective deficits (Sprangers and Schwartz 1999). In OMFS, this phenomenon may explain why patients report satisfactory long‐term outcomes following implant rehabilitation, orthognathic surgery, TMJ surgery, or oncologic reconstruction even when residual functional or esthetic impairments remain. Therefore, unchanged PROM scores should not always be interpreted as treatment failure, as they may reflect successful psychological adaptation and recalibration of patient expectations. Accounting for such cognitive adaptation demands the integration of qualitative research and real‐world patient narratives into postsurgical evaluation.

**TABLE 5:** Comparison of common patient‐reported outcome measures used in oral and maxillofacial surgery.

Instrument	Domain assessed	Strengths	Limitations	Recommended use
OHIP‐14	Oral health‐related QoL	Brief, widely validated, easy administration	Limited assessment of facial esthetics and psychosocial concerns	Implantology, general OMFS
OQLQ	Orthognathic‐specific QoL	Sensitive to facial appearance and social concerns	Limited applicability outside dentofacial deformities	Orthognathic surgery
FACE‐Q	Facial esthetics and function	Modular structure, patient‐centered design	Less long‐term validation data in OMFS	Esthetic and reconstructive facial surgery
UW‐QoL	Head and neck cancer QoL	Covers speech, swallowing, chewing, and social function	Potential ceiling effects in mild disease	Oral cancer and reconstruction
EORTC QLQ‐H&N35	Cancer‐specific QoL	Comprehensive and internationally validated	Longer administration time	Advanced head and neck cancer

Despite the methodological challenges, this body of literature demonstrates the urgent need for a new theoretical model in OMFS: the Composite Patient‐Centered Success Paradigm. This paradigm asserts that clinical success is meaningful only when it is accompanied by regained identity, dignity, social agency, and the ability to experience joy and confidence in daily life. As healthcare systems evolve toward value‐based care, reimbursement structures and clinical quality benchmarks should reflect not only what surgeons achieve technically but also how patients live afterward.

A limitation of this review is that several OMFS subspecialties, including maxillofacial trauma, cleft lip and palate surgery, medication‐related osteonecrosis of the jaw (MRONJ), and reconstruction following benign pathology, were not discussed in detail. Future studies should further explore patient‐centered outcome assessment within these important clinical domains.

The future of OMFS research and practice lies in multidisciplinary convergence. Surgical innovation must be embedded within integrated pathways involving prosthodontists, speech therapists, nutritionists, psychologists, and social workers to ensure patients regain their rightful roles in society. Introducing AI‐enabled outcome prediction, virtual expectation alignment counseling, and QoL‐focused rehabilitation models represents critical steps in modernizing the field (Zebolsky et al. 2021; Taylor et al. 2023; Barrera‐Franco et al. 2017). Recent advances in artificial intelligence have also enabled the development of predictive models for patient‐reported outcomes, automated facial analysis systems, and digital patient monitoring platforms. These technologies may facilitate personalized outcome forecasting, early identification of patients at risk of poor psychosocial adaptation, and more individualized treatment planning. As these tools continue to evolve, they may further strengthen the integration of patient‐centered care principles into OMFS practice. Only through these advancements can OMFS truly fulfill its ethical promise—not merely prolonging life or perfecting anatomy but restoring the human experience contained within every face and function we treat.

## 7 Conclusion

Success in OMFS must be defined not only by structural restoration and complication avoidance but also by the degree to which treatment enables patients to reclaim the confidence, communication, and social vitality that shape meaningful living. Across all subspecialties, this review reveals that objective improvements alone are insufficient without parallel gains in function, esthetics, emotional well‐being, and quality of life. A fully realized outcome requires integrating PROMs and QoL metrics as core indicators of success, embracing multidisciplinary rehabilitation, and aligning expectations through shared decision‐making. By redefining success as the restoration of both form and fulfillment, OMFS can more fully serve the individuals behind every surgical case.

## Author Contributions

**Betul Gedik:** methodology and draft preparation. **Abdulkadir Burak Cankaya:** data curation. **Mehmet Ali Erdem:** critical review. **Zohaib Khurshid:** conceptualization and editing. All authors have read and approved the final manuscript.

## Funding

The authors have nothing to report.

## Conflicts of Interest

The authors declare no conflicts of interest.

## Data Availability

Data sharing is not applicable to this article as no datasets were generated or analyzed during the current study.
